# Delphi Consensus on Attenuated Androgen Use for Long‐Term Prophylaxis in Hereditary Angioedema: AURA Project

**DOI:** 10.1002/clt2.70116

**Published:** 2025-11-11

**Authors:** Eunice Dias de Castro, Luís Miguel Cardoso, João Jácome de Castro, Manuel Branco Ferreira

**Affiliations:** ^1^ Allergy and Clinical Immunology Department São João Local Health Unit Porto Portugal; ^2^ Public Health and Forensic Sciences and Medical Education Department Faculty of Medicine University of Porto Porto Portugal; ^3^ Department of Endocrinology, Diabetes and Metabolism Coimbra Local Health Unit Coimbra Portugal; ^4^ i3S—Institute for Research and Innovation in Health of the University of Porto Porto Portugal; ^5^ IPATIMUP—Institute of Molecular Pathology and Immunology of the University of Porto Porto Portugal; ^6^ CIBIT—Coimbra Institute for Biomedical Imaging and Translational Research Coimbra Portugal; ^7^ Department of Endocrinology Armed Forces University Hospital Lisboa Portugal; ^8^ Immunoallergology Department Santa Maria Local Health Unit Lisboa Portugal; ^9^ Immunoallergology University Clinic Faculty of Medicine University of Lisbon Lisboa Portugal

**Keywords:** attenuated androgen, danazol, delphi, hereditary angioedema, long‐term prophylaxis

## Abstract

**Background:**

Danazol and other attenuated androgens (AAs) have been a cornerstone of Hereditary Angioedema (HAE) long‐term prophylaxis (LTP) for decades, alongside intravenous plasma‐derived C1INH (pdC1INH). Danazol's potential androgenic effects, however, present several limitations to its prescription and use. With the emergence of safer and more effective LTP drugs, guidelines are now shifting danazol to a second‐line option. These changes in HAE therapy require a new framework to guide physicians in the appropriate use of danazol in HAE LTP.

**Methods:**

This study aimed to develop a consensus on key aspects of danazol management, including discontinuation strategies, through a 2‐round Delphi methodology. Statements were defined by a steering committee of both Endocrinology and Allergy and Clinical Immunology specialists, considering the available evidence. A panel of 23 experts in HAE management voted on the statements to reach a consensus.

**Results:**

This process resulted in 46 recommendations for the prescription, monitoring, and discontinuation of danazol in LTP, proposing specific strategies for appropriate danazol use. A consensus was achieved on contraindications for danazol usage in LTP, detailed parameters for ongoing monitoring, and instructions for therapy adjustment considering treatment effect and using patient‐reported outcomes. Furthermore, seven recommendations provide guidance on the increasingly relevant challenge of danazol discontinuation in HAE patients.

**Conclusion:**

This Delphi study specifically addresses the gap in clinical guidance for danazol management in HAE patients. The resulting consensus document provides a valuable tool to aid the standardization of danazol discontinuation protocols and ensures that patients can access the safest and most effective treatment options available.

## 1 Introduction

Hereditary Angioedema (HAE) is a rare genetic disease characterized by recurrent episodes of subcutaneous and mucosal angioedema most frequently affecting the skin and the gastrointestinal and respiratory tracts. It is typically caused by a defect in the C1 inhibitor (C1INH), leading to either deficiency (HAE‐C1INH‐Type1) or dysfunction (HAE‐C1INH‐Type2) of this regulatory protein [1, 2]. HAE has an autosomal dominant inheritance pattern, although 25% of the cases arise from de novo mutations. Symptoms typically onset within the first 2 decades of life and persist throughout a patient's lifetime [3]. In Portugal, the prevalence of HAE is estimated to be 1:72,000 [4, 5, 6]. The most severe and life‐threatening manifestation of HAE is laryngeal angioedema, which has an estimated mortality risk of 8.6% due to asphyxiation, according to a systematic review [7]. HAE results from dysregulation of the kallikrein–bradykinin cascade, where C1INH impairment leads to increased kallikrein activity, causing excessive bradykinin production. This leads to vasodilation and a transient, localized increase in vascular permeability, causing swelling and inflammation [8, 9]. The unpredictable nature of these episodes contributes to significant patient anxiety, which can, in turn, trigger more attacks [9]. Moreover, HAE patients may suffer from additional comorbidities as a result of the disease itself or as side effects of their HAE treatment. Recent evidence indicates HAE patients are subject to a greater prevalence of disorders such as heart diseases and acute myocardial infarction when compared to the general population [10]. For this reason, effective preventative therapies are often essential to control the disease and improve HAE patients' quality of life (QoL), at both emotional and physical levels [9].

Management of HAE involves a combination of on‐demand treatment (ODT) for acute episodes, short‐term prophylaxis (STP), and long‐term prophylaxis (LTP) to prevent future attacks. The goals of HAE therapy are to achieve total control of the disease, or at least reduce the frequency and severity of attacks, and to normalize patients' lives [2, 11, 12]. LTP options for HAE include plasma‐derived C1‐INH (pdC1‐INH), lanadelumab, berotralstat, and attenuated androgens (AAs) [13]. AAs, used since 1960 to prevent HAE attacks, were once considered the preferred LTP owing to their accessibility and sufficient efficacy in reducing attacks [14]. However, as patients were maintained on this therapy for decades, the potential risk of AA‐related adverse events became clearer. Worldwide, danazol is one of the most used AAs for HAE long‐term prophylaxis. It is administered orally and should be used at the minimum effective dose, up to a maximum of 200 mg/day [2, 15]. Although danazol has demonstrated satisfactory success rates in preventing severe angioedema attacks at the recommended ≤ 200 mg/day, many patients respond only to higher doses of danazol, up to 600 mg/day. This heavily increases the risk of adverse events, some of them linked to danazol's dose‐dependent androgenic effect [16, 17]. Common adverse events of danazol include virilization in women, weight gain, cardiovascular disease, dyslipidemia, psychological disturbances, and hepatoxicity. Namely, comorbidities such as hypertension, diabetes and hepatic angioma occur in higher incidence in HAE patients treated with AAs than in patients never treated with androgens, as highlighted in Zanichelli et al. (2024) [10, 17].

Currently, international HAE guidelines, such as the 2021 World Allergy Organization/European Academy of Allergy and Clinical Immunology (WAO/EAACI) guideline, recommend the use of danazol only as second‐line treatment for a limited subset of patients, with pdC1‐INH, lanadelumab, and berotralstat being preferred first‐line LTPs. It is recommended that the use of danazol be regarded critically, considering its androgenic and anabolic effects, drug interactions, and contraindications, with its dosage adjusted according to clinical response and always aiming for the minimum effective dose [2]. Most patients currently treated with danazol started this therapy before the emergence of newer LTP options and have been on treatment for several years. However, with this paradigm shift, the maintenance of danazol therapy in these patients is being reconsidered. In many cases, physicians and patients choose to continue danazol due to the drug's accessibility and their familiarity with its use [18]. Moreover, access to first‐line LTPs is still limited in countries with lower‐resources for HAE management [19, 20]. Nonetheless, concerns over danazol's long‐term side effects may prompt the decision to switch the therapy. This decision is complicated by the potential for withdrawal symptoms and rebound HAE attacks, which can make discontinuation a challenging process [18, 21, 22]. Furthermore, while international guidelines emphasize newer treatments, there is a significant gap in evidence‐based practical strategies for tapering or discontinuing more outdated LTPs, such as danazol. Currently, there are no established guidelines or large‐scale studies to guide physicians on this subject. Thus, as the paradigm of HAE treatment evolves, it is essential to develop a consensus that not only supports the prescription and monitoring of danazol but also guides its discontinuation in clinical practice.

The “AURA—Attenuated Androgen Use Reduction in the Management of Hereditary Angioedema” project aims to develop a consensus document on the appropriate management of AAs, specifically danazol, for LTP in HAE patients through a 2‐round Delphi process with allergists and clinical immunologists experienced in the management of HAE.

## 2 Methods

### 2.1 Delphi Methodology

Allergy and clinical immunology specialists (ACIs) were asked to evaluate statements on the management of LTP with danazol in patients with HAE through an online Delphi process over a period of 2 months [23, 24]. This methodology was selected because it ensures the retrieval of unbiased feedback to reach consensus on complex topics and allows the participation of experts from different locations within a larger timeframe.

The consensus methodology was based on two rounds, with topics defined and reviewed by a Steering Committee (SC) composed of 2 ACIs with extensive expertise in HAE and two endocrinology specialists, knowledgeable of HAE and with extensive expertise in androgen therapy. SC members did not participate in the rounds. The 2 Delphi rounds were executed through online questionnaires using the Qualtrics platform and were distributed via direct link to the participants. The SC was responsible for outlining the topics to evaluate regarding danazol use in LTP of HAE, drafting and revising the statements proposed on round 1 and 2, respectively, and analyzing the results and feedback provided after each round. The SC did not receive input from the sponsor in this process.

### 2.2 Literature Review and Statement Formulation

A targeted exploratory literature review was conducted between June and July 2024 to support the formulation of the statements used in the Delphi. The review included an extensive search in PubMed, with its scope including guidelines for the treatment and monitoring of HAE, studies on the effects of AAs and danazol, and reports on HAE patients under danazol treatment and discontinuation. The search terms included combinations of: “hereditary angioedema”, “danazol”, “attenuated androgen”, “long‐term prophylaxis”, “discontinuation”, and “adverse events”. Given the long‐standing use of danazol in HAE and the limited evidence on this topic, the search included articles dating back from 1970 until August 2024. The review was not systematic, and no formal inclusion or exclusion criteria were predefined. Instead, references were selected based on their relevance and contribution to the formulation of evidence‐based and context‐appropriate statements for expert evaluation.

Based on the evidence collected in the literature review, statements were developed and discussed between the SC until a final selection was reached. The final selection was organized into four main topics (therapeutic decisions, monitoring of patients, modification, and discontinuation of LTP with danazol), comprising 46 statements. Among them, 2 address a list of monitoring tests: one with 3 clinical examinations and another with 7 laboratory analyses. To simplify the survey, each clinical and laboratory test was presented as an individual statement in the first round, resulting in a total of 54 initial statements.

### 2.3 Delphi Setup and Consensus

The Delphi participants were selected in collaboration with SPAIC (Portuguese Society of Allergology and Clinical Immunology), who identified 28 specialists with HAE experience from different regions of Portugal. Of these, 23 experts agreed to participate and answered both rounds.

In the survey, the participants were asked to rate each statement on a 3‐level scale, with (1) “Strongly disagree”, (2) “Agree, with comments” or (3) “Strongly agree”. Quotes with supporting evidence and their sources were provided for each statement. Responses rated as level 1 or 2 required participants to submit their feedback via open‐ended text boxes. The use of a three‐point scale, supported by previous Delphi studies, was elected to simplify decision‐making and encourage participation without overburdening the experts, given the limited number of rounds, the relatively small expert panel and the high volume of statements to evaluate [25, 26]. The incorporation of mandatory qualitative feedback for any response indicating disagreement or partial agreement ensured that no clinical nuance was lost, allowing for a more detailed analysis of expert input.

The Delphi was conducted in two rounds. Round 1 allowed for an initial assessment of all 54 statements, and Round 2 focused on re‐evaluating statements that did not achieve consensus in the first round. Consensus was defined as over 85% of participants selecting “Strongly Agree.” Statements meeting this threshold were added to our final list of recommendations. Results were analyzed in Excel, and all open‐ended feedback was collected for consideration by the SC during the preparation of Round 2 and the development of the manuscript.

## 3 Results

**FIGURE 1:**
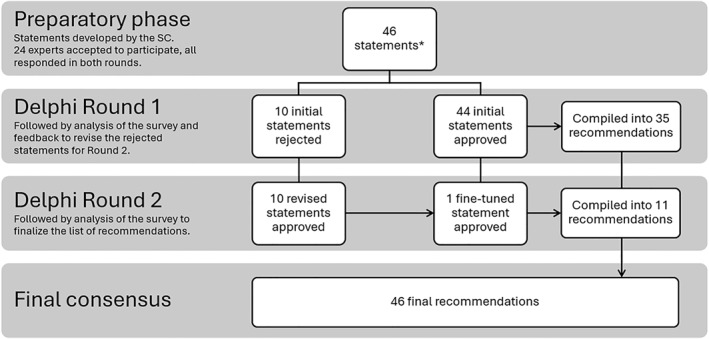
Breakdown of the AURA Delphi process. (*) Out of the 46 proposed statements, 2 referred to monitoring tests: one with 3 clinical examinations and another with 7 laboratory analyses. To simplify the survey, each clinical and laboratory test was presented as an individual statement in Round 1, leading to a total of 54 initial statements. Since all 10 individual monitoring test statements were approved in Round 1, they were compiled into their 2 respective recommendations, together with the other 33 statements approved in the first round, making a total of 35.

In this study, 23 allergists and clinical immunologists participated in 2 Delphi rounds, concluding the process with a 100% retention rate between rounds. A consensus was reached in all 46 statements, with a 100% statement approval rate after Round 2 (Figure 1). These recommendations were divided into 4 main topics: therapeutic decisions for patients with HAE (Table 1), monitoring of patients with HAE on LTP with danazol (Table 2), modification of LTP with danazol in patients with HAE (Table 3), and discontinuation of LTP with danazol in patients with HAE (Table 4).

**TABLE 1:** Recommendations regarding the therapeutic decisions in patients with HAE.

Recommendations	Consensus (%)
Therapeutic decisions for long‐term prophylaxis with danazol in patients with HAE	
1. In long‐term prophylaxis, the average daily dose of danazol should not exceed 200 mg. In particular situations, doses of up to 600 mg/day may be considered, for the shortest possible time.	87
2. Danazol should only be considered as second‐line treatment in LTP, where specific therapies for HAE are available for this indication.	91
Therapeutic decisions in patients with HAE: Absolute contraindications for LTP with danazol	
3. Female patients who intend to become pregnant within 1 year, or who are already pregnant or breastfeeding.	96
4. Patients with cancer, namely hormone‐dependent such as breast, ovarian, prostate, liver, or pancreatic cancer.	100
5. Patients with a history of ischemic heart disease.	100
6. Patients with thrombotic risk.	91
Therapeutic decisions in patients with HAE: Relative contraindications for LTP with danazol	
7. Children and adolescents in pubertal development (Tanner stages I‐IV).	96
8. Male patients of reproductive age who intend to have children, if fertility assessment shows low sperm count.	100
9. Female patients.	87
10. Patients on a therapeutic regimen at risk of interaction with danazol.	96
11. Patients with migraine, especially if not under control.	87
12. Patients with hypertension, especially if not under control.	87
13. Patients with dyslipidemia, especially if not under control.	91
14. Patients with diabetes, especially if not under control	96
15. Patients with hepatic abnormalities, such as elevated transaminases.	91
16. Patients with structural liver disease of unknown origin, including de novo liver nodules.	87
17. Patients with a body Mass Index ≥ 35 or with complications from obesity.	96
18. Patients with polycythemia and thrombocytosis.	91
19. Patients with severe acne and refractory to dermatologist‐optimized therapy.	96
20. Patients with androgenic alopecia with a significant impact on quality of life and after a risk‐benefit assessment by a dermatologist	100
21. Patients with psychiatric pathology susceptible to decompensation with androgenic therapy.	100

Abbreviations: HAE: hereditary angioedema; LTP: long‐term prophylaxis.

**TABLE 2:** Recommendations regarding monitoring of patients with HAE on LTP with danazol.

Recommendations	Consensus (%)
Monitoring of patients with HAE on LTP with danazol	
22. Clinical evaluation with weight^a^ and blood pressure^a^ measurement, and signs of virilization^a^ analysis at least once a year.	
a. Weight^a^	91
b. Blood pressure^a^	87
c. Signs of virilization^a^	87
23. Laboratory evaluation (including blood count^a^, blood glucose^a^, AST, ALT, GGT^a^, lipid profile^a^, creatine phosphokinase^a^, urinalysis^a^ and alpha‐fetoprotein^a^) at least once a year.	
d. Blood count^a^	100
e. Blood glucose^a^	91
f. AST, ALT, GGT^a^	96
g. Lipid profile^a^	96
h. Creatine phosphokinase^a^	100
i. Urinalysis^a^	100
j. Alpha‐fetoprotein^a^	96
24. Abdominal (liver) ultrasound before starting therapy, and every 6 months if treated with daily doses of danazol of > 200 mg or once a year with daily doses ≤ 200 mg.	100
25. Assessment of disease control, through the angioedema control test (AECT), ideally at each appointment, at least once a year.	91
26. Assessment of the impact of HAE on the patient's quality of life, through the angioedema quality of life questionnaire (AE‐QoL), ideally at each appointment, at least once a year.	91
27. Assessment of the evolution of the patient's mental state at each appointment, namely mood swings, changes in libido, aggressiveness, anxiety and depression.	91
28. Assessment of patient satisfaction with the therapy at each appointment.	100
29. Monitoring at each appointment for self‐management of medication (questioning the patient about dosage frequency and raising awareness of the risks of increasing the dose).	100

Abbreviations: HAE: hereditary angioedema; LTP: long‐term prophylaxis.

^a^ Statements evaluated separately in the Delphi survey.

**TABLE 3:** Recommendations regarding modification of LTP treatment with danazol in patients with HAE.

Recommendations	Consensus (%)
Modification of LTP with danazol in patients with HAE: Reasons to trigger a dose reduction or discontinuation	
30. Patient with obvious signs of virilization (e.g. voice changes, hirsutism, alopecia, breast atrophy) that persist even with a decrease in the dose of danazol.	91
31. Patient with recurrent and disabling myalgias, with no other apparent cause.	96
32. Patient with clinically significant weight gain in the last year on danazol therapy, after exclusion of other secondary etiologies, weight progression and/or lack of response to lifestyle measures and therapies for obesity control.	96
33. Patient presents relevant and persistent changes in their psychic state, such as aggressiveness, anxiety and depression, with interference in personal or family life.	87
34. Patient remains controlled and without angioedema crises, preferably with a score greater than or equal to 12 in the AECT in the last year.	91
35. Patient develops an absolute contraindication.	96
36. Patient develops a relative contraindication.	87
Modification of LTP with danazol in patients with HAE: Reasons to trigger a treatment readjustment	
37. Patient has a score below 10 in the AECT in two consecutive appointments.	87
38. Patient has persistently higher than 39 on AE‐QoL (defined as moderate to high impact) in the past 12 months.	96
39. Patient remains with 1 or more attacks per month and/or has a severe laryngeal or abdominal attacks in the last 12 months, despite complying with the prescribed therapy.	100

Abbreviations: HAE: hereditary angioedema; LTP: Long‐term prophylaxis.

**TABLE 4:** Recommendations regarding discontinuation of LTP with danazol in patients with HAE.

Recommendations	Consensus (%)
Discontinuation of LTP with danazol in patients with HAE	
40. The danazol discontinuation process should follow a personalized approach based on disease severity, duration of therapy, adverse events experienced, and patient preferences.	100
41. The process of discontinuation of danazol should be carefully monitored for increased frequency and severity of angioedema attacks.	96
42. The patient should be monitored for any signs of androgen insufficiency (such as clinically significant mood swings, changes in libido, lack of energy, and loss of muscle strength) during the danazol discontinuation process.	100
43. Discontinuation of danazol should be done gradually.	87
44. Gradual discontinuation of danazol should preferably be done by reducing the dose, and, if not possible, by reducing the frequency of dosing.	91
45. If acute attacks of angioedema occur during the process of reducing danazol dosage due to a relative contraindication, consider switching to another LTP, or resuming the previous dose when the average daily dose of danazol does not exceed 200 mg.	96
46. If acute attacks of angioedema occur during the process of reducing danazol dosage due to an absolute contraindication, consider switching to another LTP.	100

Abbreviations: HAE: hereditary angioedema; LTP: Long‐term prophylaxis.

In Round 1, open from August 5th to September 1st, 2024, 54 statements were evaluated. Once completed, 44 statements had been approved (i.e., 85% of “Strongly agree” responses) including all 10 statements regarding individual clinical and laboratory tests. These were combined into 2 final recommendations, one for the 3 approved clinical assessments and another for 7 approved laboratory evaluations (statements 22 and 23, see Table 2). Therefore, a total of 35 statements were approved in Round 1. Ten statements did not reach consensus in Round 1 and were revised considering participants' feedback. Additionally, the SC identified 1 statement approved that required fine‐tuning. Hence, 11 statements were subject to further evaluation (see Figure 1). Round 2 occurred between September 6th and 24th. All statements in Round 2 were approved, making a final total of 46 recommendations.

### 3.1 Therapeutic Decisions for Long‐Term Prophylaxis With Danazol in Patients With Hereditary Angioedema

Regarding therapeutic decisions for danazol LTP in HAE patients, namely the statements on its prescription, 21 recommendations were approved (Table 1). Statements 1, 2, 7, 19, 20, and 21 did not achieve the 85% consensus threshold in Round 1 and were therefore rejected and revised by the SC for Round 2. Statement 3 was approved in Round 1 but was reassessed by the SC taking into account the participant's feedback and a refined version was proposed for Round 2 (Supporting Information S1: Table 1). These 7 recommendations were approved in Round 2.

### 3.2 Monitoring of Patients With Hereditary Angioedema on Long‐Term Prophylaxis With Danazol

All statements concerning danazol monitoring in HAE LTP were approved in Round 1, culminating in 8 recommendations (Table 2). Recommendations 22 and 23 were evaluated separately as statements 3 and 7, respectively, to allow voting for each specific assessment. Since all were approved, they were combined into their respective recommendations.

### 3.3 Modification of Long‐Term Prophylaxis With Danazol in Patients With Hereditary Angioedema

A total of 10 recommendations were reached for the modification of danazol LTP in HAE patients (Table 3). These recommendations clarify the situations that elicit danazol dose reduction or discontinuation and the ones that require treatment readjustment. Recommendations 32, 38, and 39 were rejected in Round 1 and approved in Round 2 after the statements were fine‐tuned based on the panel's feedback (Supporting Information S1: Table 1).

### 3.4 Discontinuation of Long‐Term Prophylaxis With Danazol in Patients With Hereditary Angioedema

Seven recommendations were approved regarding the approach to danazol discontinuation (Table 4) with only one requiring revision in Round 2 (Recommendation 45, Supporting Information S1: Table 1).

## 4 Discussion

While newer LTP options for HAE, such as lanadelumab, berotralstat, and pdC1‐INH, have shifted the treatment paradigm in higher‐resource countries, access to these therapies remains limited in numerous regions due to regulatory, logistical, or financial constraints [15, 19, 20]. In such contexts, second‐line LTPs, namely danazol, continue to play a central role in HAE management and the transition to first‐line options is delayed. Our Delphi consensus seeks provide practical guidance on danazol prescription, monitoring, and treatment modification, especially for clinicians operating in settings where AAs remain a primary LTP option and where experience in danazol discontinuation is low.

### 4.1 Therapeutic Decisions for Long‐Term Prophylaxis With Danazol in Patients With Hereditary Angioedema

The goal of LTP in HAE is to normalize a patient's life. Treatment should be patient‐specific, personalized to disease activity, available resources, and the burden of therapy [2]. Our consensus on danazol prescription is aligned with other international guidelines, which recommend that danazol use should not exceed 200 mg/day and should only be considered as second‐line LTP (Recommendations 1 and 2) [2, 11]. The recommendation that danazol prescription should always aim for the minimum effective dose that achieves disease control is well‐established internationally. As danazol doses above the recommended threshold are associated with an increased risk of androgen‐related toxicities, doses between 200 and 600 mg/day are discouraged and may only be considered in exceptional situations and for the shortest possible duration. Long‐term exposure to higher danazol doses is associated with increased likelihood of serious complications, particularly in patients with pre‐existing risk factors, as incidence and severity of danazol‐related adverse events are dose‐dependent, therefore, a modification of LTP may need to be considered in these scenarios [2, 10, 17, 27]. Our expert panel highlighted the importance of individualized assessment, taking into account each patient's medical history and risk profile.

Regarding contraindications to danazol use, the panel unequivocally agreed with some precautions that align with the strong evidence of danazol‐mediated adverse events. Namely, we highlight the absolute contraindication of danazol to female patients planning to conceive within 1 year (Recommendation 3) and relative contraindication to male patients when fertility may be impaired (Recommendation 8). These contraindications reflect a heightened awareness of danazol's reproductive risks, and while it is well established that danazol exposure during pregnancy can cause virilization in a female fetus, our expert panel also considered evidence suggesting that high androgen levels may diminish fertility in both men and women [28, 29, 30]. Moreover, unlike existing guidelines that primarily focus on pregnant or lactating women, our consensus also designates all female patients, in general, as a relative contraindication (Recommendation 9), due to danazol's significant risk of virilizing side effects [17, 31, 32].

Recent data suggests that HAE itself is associated with endothelial dysfunction, even in young C1‐INH HAE patients without signs of atherosclerotic disease, which further highlights the cardiovascular vulnerability in this population [33]. Therefore, cardiovascular risks associated with danazol were another critical area of consensus, particularly in patients with a history of ischemic heart disease or a higher risk for thrombotic events. Dyslipidemia, hypertension, polycythemia, thrombocytosis, and a higher risk of ischemic events have all been linked to danazol use [10, 17, 34, 35, 36, 37, 38, 39, 40]. These phenomena can increase the risk of thrombosis, ischemic heart disease, and heart failure. Therefore, it is recommended that patients with these risk factors should avoid danazol (Recommendations 5, 6, 12, 13, and 18). The panel also endorsed that cancer, especially if hormone‐dependent, should be an absolute contraindication for danazol treatment (Recommendation 4), as there is evidence for the detrimental correlation between danazol exposure and ovarian, prostate, and liver cancer [41, 42, 43].

Drug interactions were also flagged as major concern. Experts strongly agreed that the patient's ongoing therapeutic regimens should be evaluated, and danazol must be avoided when there is a risk of interaction with pre‐existing medications (Recommendation 10). The medications with higher drug–drug interaction risk include anticoagulants, statins, antidepressants, antiepileptic drugs, antidiabetics, and immunosuppressant drugs [14, 44].

Likewise, consensus was reached regarding the relative contraindication for danazol use in pediatric patients between Tanner stages I to IV (Recommendation 7), due to its potential effects on pubertal development [11, 17, 45]. This is aligned with current international guidelines that advise against the use of LTP with AAs in pediatric patients prior to Tanner stage V unless strictly necessary, in which case patients should be regularly monitored and for any side effects and managed at the minimum effective dose [2, 11, 46].

The expert panel also identified several specific health conditions where danazol use should be avoided, emphasizing the risk of side effects that are often underrepresented in current guidelines. Headaches are a commonly reported side effect and have been referred by HAE patients as a reason for danazol discontinuation [17, 22, 39, 47]. Danazol has also been found to cause hyperglucagonemia, hyperglycemia, and hyperinsulinemia, which can be harmful to patients with diabetes and should be avoided in these patients [39, 48, 49, 50]. Additionally, due to the common side effect of weight gain [22, 47, 51], the experts agreed that patients with obesity and obesity‐related complications should avoid danazol. Finally, due to the possible detrimental effects of AAs on acne, alopecia, and psychological disorders (e.g., anxiety, depression, mood changes), experts agreed that patients severely affected and for these conditions should avoid danazol as well [17, 31, 39, 47, 52, 53]. The panel reached a consensus on the relative contraindication of danazol for these diseases when not controlled by the best medical practices (Recommendations 11, 13, 14, 17, 19, 20, and 21). This recommendation highlights the importance of understanding how danazol may impact seemingly unrelated conditions.

Another major concern with danazol use is its potential to cause hepatic toxicity. Evidence shows that danazol use can lead to elevations in serum aspartate aminotransferase (AST) and alanine aminotransferase (ALT) levels, which may indicate liver stress or injury. This has been demonstrated to be a dose‐dependent effect, also aggravated by the duration of exposure to danazol [17]. Moreover, some patients under long‐term treatment with danazol developed hepatocellular adenomas and even carcinoma [39, 43, 49, 54]. Consequently, standard monitoring for patients on danazol includes regular liver function tests and hepatic ultrasounds [2]. Given these risks, the panel agreed that danazol should be avoided in patients with pre‐existing hepatic abnormalities (Recommendation 15 and 16).

### 4.2 Monitoring of Patients With Hereditary Angioedema on Long‐Term Prophylaxis With Danazol

As with any pharmacological treatment, the effectiveness and safety of danazol must be regularly assessed. Given the known risks associated with danazol, particularly with long‐term use, close monitoring is essential [2, 14].

It is well established that treatment with danazol requires regular clinical and laboratory monitoring [2, 11]. The expert panel agreed that at least once a year, signs of weight gain, hypertension, and virilization should be monitored, and biochemical testing, including hemogram, glycemia, liver function tests, lipid profile, and urine tests, should be performed as well [17, 38, 39, 55, 56, 57]. Additionally, regular liver ultrasounds are mandatory, with their frequency depending on the dose of danazol prescribed [54]. These recommendations are well‐aligned with the current guidelines, with the panel underscoring the importance of a regular check‐up (Recommendations 22, 23, and 24).

The panel also emphasized the need to monitor patient‐reported outcomes during danazol LTP. Incorporating tools like the Angioedema Control Test (AECT) and the Angioedema Quality of Life Questionnaire (AE‐QoL) into follow‐up appointments is strongly recommended by current guidelines (Recommendations 25 and 26). These tools provide objective measures of treatment success and help assess disease control and quality of life [2].

Finally, the consensus endorses that mental health concerns should be appropriately managed (Recommendation 27). As chronic exposure to danazol may worsen the psychological and behavioral state of patients (increasing insomnia, agitation, mood swings, anxiety, depression, and aggressiveness) physicians must be aware of these aspects [17, 52, 54]. Regular discussions about patient satisfaction are also critical to evaluate whether the treatment is meeting both the patient's and physician's goals, and whether therapy adherence is maintained (Recommendations 28 and 29). If patients feel unsatisfied or experience worsening symptoms, they may attempt to self‐manage their medication, which can be harmful if unnoticed [11, 17, 27].

### 4.3 Modification of Long‐Term Prophylaxis With Danazol in Patients With Hereditary Angioedema

Although danazol discontinuation has become an increasingly discussed topic in recent years, there is little information regarding how and when a patient's danazol therapeutic strategy should be re‐evaluated.

The panel of experts reached consensus on several scenarios where danazol discontinuation or, at least, a dosage reduction is warranted. First, the development of new conditions or adverse events associated with danazol that significantly impact the patient's QoL, such as signs of virilization, disabling myalgias, weight gain, and alterations in the psychological state should trigger a reassessment of therapy (Recommendations 30, 31, 32, 33, 35 and 36) [17, 22, 31, 39, 47, 51, 52, 53]. Secondly, in line with international recommendations, the panel agreed that therapy modification should be considered for patients who have been stable without HAE attacks for an extended period (Recommendation 34). To ensure greater confidence in disease stability and provide a measurable benchmark, the recommendation suggests that an AECT score of at least 12 maintained for one year be considered as the threshold for the reduction or discontinuation of danazol LTP physicians [2, 11]. Knowing an AECT score ≥ 10 is the validated cutoff for well‐controlled disease, this cautious approach proposed by the SC and approved by the Delphi panel aims to avoid premature dose adjustments in patients whose scores may fluctuate near the lower boundary of control [58].

Regarding scenarios that may warrant a readjustment of therapy, the panel agreed that signs of uncontrolled disease or worsening quality of life, whether indicated by an increase in HAE attacks or detected through tools like the AECT and AE‐QoL, should prompt an increase in danazol dosage (if not surpassing the maximum recommended dose of 200 mg/daily) or a switch to a new LTP drug (Recommendations 37, 38 and 39) [11, 22, 58, 59, 60].

### 4.4 Discontinuation of Long‐Term Prophylaxis With Danazol in Patients With Hereditary Angioedema

The process of danazol discontinuation has become an increasingly discussed topic in recent years. However, there is still debate regarding the best approach to this process [18, 61]. Studies on this topic, although extremely valuable, are often limited by small sample sizes and significant variability between subjects [21, 22, 61]. Furthermore, patients commonly exhibit significant anxiety with the AA discontinuation process, many not properly following the scheduled protocol and returning to previous danazol dosages in response to rebound HAE attacks [18, 22, 61, 62]. Research is further challenged by the rarity and chronicity of the disease, as there are few patients, sometimes with decades‐long history of danazol treatment [18, 22]. Even in patients who appear to tolerate danazol without apparent adverse events, the risks associated with prolonged exposure should not be overlooked, as longstanding use of danazol increases the risk of side effects [17]. Therefore, in the long term, stopping danazol or switching LTP altogether can be beneficial for all danazol‐treated patients.

The expert panel emphasized the importance of tailoring the discontinuation protocol to each patient's profile and preferences and closely monitoring disease activity throughout the process (Recommendations 40 and 41) [18, 21, 22]. Proper supervision of the process, with consideration of the patient's needs and expectations, may reduce the likelihood of premature withdrawal from the discontinuation process. The panel also recommended evaluating patients for signs of androgen insufficiency during danazol discontinuation (Recommendation 42). Prolonged exposure to danazol can suppress the hypothalamic‐pituitary‐gonadal axis and may lead to withdrawal symptoms, such as anxiety, depression, mood swings, fatigue, muscle loss, headache, sexual dysfunction, and impaired cognition [21].

As for the timeline of discontinuation, the experts agreed that a gradual tapering process is preferred (Recommendation 43). This approach is supported by recent international guidelines and case reports [11, 21, 22]. While abrupt cessation may be necessary in certain circumstances, such as an unexpected pregnancy, gradual tapering is generally considered safer and less likely to result in adverse events [22]. Experts also reached consensus on the recommendation that the tapering process should preferably be done by reducing the danazol dose, or a reduction in frequency if dose reduction is not possible (Recommendation 44). Johnston et al. (2021) propose that dose reduction be performed in 50 mg/day increments, in intervals sufficient to assess if disease control has declined. For reduction in frequency, the authors' suggest that daily doses are switched for every other day for a few weeks, followed by every third day for a few weeks and then cessation of danazol. In both scenarios, the availability of ODT is case of relapse is emphasized [21].

If HAE attacks increase during the danazol tapering process, the panel agreed that the discontinuation protocol should be readjusted. If the cause for discontinuation is a relative contraindication, switching to a newer LTP alternative should be considered, particularly in patients at risk of androgen‐related adverse events. Alternatively, resuming the previous danazol dose, provided it does not exceed 200 mg/day, may be acceptable in selected cases (Recommendation 45). If the cause is an absolute contraindication, transitioning to another LTP is mandatory, as it will allow continuous disease control while minimizing the risks associated with danazol (Recommendation 46). This consensus emphasizes the balance between effective prophylaxis and patient safety and promotes a more standardized strategy for danazol tapering.

Overall, this consensus demonstrates a strong alignment between physicians on the topic of danazol use in patients with HAE, as all final recommendations were approved after two rounds. Our approach has some limitations, including the low availability of recent large‐scale studies on the effects of danazol in HAE patients to help support the formulation of the statements as well as the risk of interaction between participants outside of the structured Delphi process. Additionally, the use of a three‐point scale may have limited the granularity of expert responses. However, this format was deliberately chosen to simplify decision‐making and support full participation, given the number of statements and the two‐round format. To minimize loss of nuance, mandatory qualitative feedback was collected for all responses without full agreement and carefully analyzed by the SC, which led to revisions in several statements and ultimately contributed to the 100% approval rate. Notably, this consensus allowed the integration of the perspective of various HAE specialists across Portugal, a key strength of the Delphi method. This study demonstrates that experts are well‐aligned their approach to AA use in LTP in HAE patients in a setting where danazol still plays a significant therapeutic role.

While our conclusions align with established international guidelines, they also extend further the existing recommendations by including new considerations on the prescription, monitoring, and modification of danazol therapy. Regarding the future of danazol, recent studies have highlighted a growing trend toward reducing danazol use for LTP, driven by the introduction of newer therapies with fewer side effects [2, 18, 21]. Namely, in the United States, danazol prescriptions for LTP are declining significantly, dropping from 56% of most used HAE therapies in 2010 to just 6% in 2019 [15]. Still, it is important to acknowledge that the transition away from older generation LTPs is not occurring uniformly across the world. In many healthcare systems, newer therapies may not yet be available, affordable, or reimbursed, meaning that danazol continues to be a key player in disease management in many countries. Feedback provided by the experts in our Delphi survey support this notion, as some stated that the high cost of other LTPs might influence the preference for danazol in certain situations.

New LTPs, such as pdC1‐INH, lanadelumab and berotralstat, offer patients safer alternatives with fewer adverse effects, making them a first‐line option in opposition to danazol [2]. Berotralstat presents another daily oral option for LTP, with fewer side effects, while pdC1‐INH and lanadelumab are subcutaneous injectable treatments, usually administered 2 times a week and once every 2 weeks, respectively, where a dose reduction to 300 mg every 4 weeks may be considered for patients on lanadelumab treatment who are stably attack free [2, 18]. Other LTP options are currently under development as well, with more oral and injectable options being tested in ongoing trials [13, 63]. The next step is clarifying the best approach to transitioning to these safer LTP alternatives. It is expected that ongoing projects such as the SHAERPA (Stopping androgen treatment in patients with HAE—characterization of reasons and protocols and development of advice for patients and physicians) project will provide the evidence needed to standardize this process and better anticipate the challenges [18].

## 5 Conclusion

Although danazol has served as a cornerstone of HAE prophylaxis for several decades, the emergence of safer and more effective LTP options is leading to a re‐evaluation of danazol use. This is, to our knowledge, the first Delphi study specifically addressing the gap in clinical guidance for danazol management in HAE patients. It provides 46 recommendations on the prescription, monitoring, modification, and discontinuation of danazol. The recommendations emphasize ongoing risk assessment, personalized treatment plans, and patient‐reported outcomes to guide therapeutic decisions. This consensus document provides a tool to aid the standardization of danazol discontinuation protocols and ensure that patients can access the safest and most effective treatment options available.

## Author Contributions

**Eunice Dias de Castro:** conceptualization, methodology, validation, writing – review and editing, writing – original draft, supervision. **Luis Miguel Cardoso:** conceptualization, validation, methodology, writing – review and editing, writing – original draft. **Joao Jacome de Castro:** conceptualization, methodology, validation, writing – review and editing, writing – original draft. **Manuel Branco Ferreira:** conceptualization, methodology, validation, writing – review and editing, writing – original draft, supervision.

## Conflicts of Interest

E.D.C. has received advisory board and/or lecture fees from Takeda, Kalvista, CSL Behring and Biocryst and congress support from Takeda and CSL Behring. L.M.C. has received travel grants from Recordati and consulting and speaker fees from Recordati and Takeda. J.J.C. has received fees as a consultant or speaker from Abbott Diagnostics, AstraZeneca, Bayer, BIAL, Boehringer Ingelheim, GlaxoSmithKline, Lilly, Medinfar, Menarini, Menarini Diagnostics, Merck Serono, MSD, Novartis, Novo Nordisk, Recordati, Roche Diagnostics, Sanofi and Takeda. M.B.F. has received advisory board and/or lecture fees from Takeda, CSL Behring and Biocryst and congress support from Takeda.

## Supporting information


Supporting Information S1

## Data Availability

The data that support the findings of this study are available from the corresponding author upon reasonable request.
