# Supplementary material for: Delphi Consensus on Attenuated Androgen Use for Long‐Term Prophylaxis in Hereditary Angioedema: AURA Project
Source: Clin Transl Allergy. 2025 Nov 11;15(11):e70116. doi: 10.1002/clt2.70116 (PMC12606019; doi:10.1002/clt2.70116)
Supplement: Supplementary file 1 — Supporting Information S1 [file CLT2-15-e70116-s001.docx]

# Supplementary Material

Supplemental Table 1: Statements rejected or revised after Round 1.

| **Rejected Statements** | | **Consensus (%)** |
| --- | --- | --- |
| **Therapeutic Decisions for Long-Term Prophylaxis with Danazol in Patients with HAE** | | |
| 1. **[Recommendation 1]** In long-term prophylactic treatment, the daily dose of danazol should not exceed 200 mg. | | 78 |
| 1. **[Recommendation 2]** Danazol should only be considered as a second-line treatment in long-term prophylactic treatment. | | 83 |
| **Therapeutic Decisions in Patients with HAE:**  **Relative Contraindications for LTP with Danazol** | | |
| 1. **[Recommendation 3]** Female patients who intend to become pregnant within one year, or who are already pregnant | |  |
| 1. **[Recommendation 7]** Patients in puberty. | | 78 |
| 1. **[Recommendation 19]** Patients with severe acne. | | 83 |
| 1. **[Recommendation 20]** Patients with alopecia. | | 70 |
| 1. **[Recommendation 21]** Patients under psychiatric care, after discussion with their psychiatrist. | | 78 |
| **Modification of LTP with Danazol in Patients with HAE:**  **Dose Reduction or Discontinuation** | | |
| 1. **[Recommendation 32]** Patient with clinically significant weight gain in the last year. | | 74 |
| **Modification of LTP with Danazol in Patients with HAE:**  **Treatment Readjustment** | | |
| 1. **[Recommendation 38]** Patient scores higher than 39 on the AE-QoL (defined as moderate to high impact) in two consecutive appointments. | | 83 |
| 1. **[Recommendation 39]** Patient remains with > 1 attacks per month in the last 12 months, despite complying with the prescribed therapy | | 83 |
| **Discontinuation of LTP with Danazol in Patients with HAE** | | |
| 1. **[Recommendation 45]** If acute attacks of angioedema occur during the process of reducing danazol dosage: consider returning to the previous dose (when the reason for discontinuation of danazol is a relative CI). | 83 | |

HAE: Hereditary Angioedema; LTP: Long-term Prophylaxis

Supplemental Table 2: List of experts of the “AURA - Attenuated Androgen Use Reduction in the Management of Hereditary Angioedema” project, who participated in the Delphi.

| **Name** | **Affiliation** |
| --- | --- |
| Amélia Spínola Santos | ULS Santa Maria |
| Ana Luisa Pinhal | ULS São João |
| Ana Margarida Romeira | ULS São José |
| Ana Morete | ULS Aveiro |
| Anabela Lopes | ULS Santa Maria |
| Carlos Lozoya | ULS da Cova da Beira |
| Célia Costa | ULS Santa Maria |
| Celso Pereira | ULS Coimbra |
| Daniel Machado | ULS do Alto Minho |
| Frederico Regateiro | ULS Coimbra |
| Graça Loureiro | ULS Coimbra |
| Joana Caiado | ULS Santa Maria |
| Joana Cosme | ULS Santa Maria |
| Joana Queirós Gomes | ULS Gaia e Espinho |
| João Marcelino | ULS Arrábida |
| Margarida Mesquita | ULS de Braga |
| Mariana Bragança | ULS São João |
| Natacha Santos | ULS do Algarve |
| Paula Alendouro | ULS do Alto Ave |
| Paula Leiria Pinto | ULS São José |
| Rodrigo Rodrigues Alves | Hospital do Divino Espírito Santo de Ponta Delgada |
| Rui Silva | ULS Trás-os-Montes e Alto Douro |
| Sofia Ferreira | Hospital Dr. Nélio Mendonça |
